# Differential neural processing of unpleasant sensory stimulation in patients with major depression

**DOI:** 10.1007/s00406-020-01123-0

**Published:** 2020-04-11

**Authors:** Kathrin Malejko, Rebecca C. Brown, Paul L. Plener, Martina Bonenberger, Heiko Graf, Birgit Abler

**Affiliations:** 1 Department of Psychiatry and Psychotherapy III, Ulm University, Ulm, Germany; 2 Department of Child and Adolescent Psychiatry and Psychotherapy, Ulm University, Ulm, Germany; 3 Department for Child and Adolescent Psychiatry, Medical University of Vienna, Vienna, Austria

**Keywords:** Unpleasant sensory stimulation, Depression, fMRI, Electric stimulation, Somatosensory processing

## Abstract

An altered processing of negative salient stimuli has been suggested to play a central role in the pathophysiology of major depression (MD). Besides negative affective and social stimuli, physical pain as a subtype of negative sensory stimulation has been investigated in this context. However, the few neuroimaging studies on unpleasant sensory stimulation or pain processing in MD report heterogeneous findings. Here, we investigated 47 young females, 22 with MD and 25 healthy controls (HC) using fMRI (3.0 T). Four levels of increasingly unpleasant electrical stimulation were applied. Ratings of stimulus intensity were assessed by a visual analogue scale. fMRI-data were analyzed using a 2 × 4 ANOVA. Behavioral results revealed no group differences regarding accuracy of unpleasant stimulation level ratings and sensitivity to stimulation. Regarding neural activation related to increasing levels of unpleasant stimulation, we observed increasing activation of brain regions related to the pain and salient stimulus processing corresponding to increasingly unpleasant stimulation in controls. This modulation was significantly smaller in MD compared to controls, particularly in the dorsal anterior cingulate cortex, the somatosensory cortex, and the posterior insula. Overall, brain regions associated with the processing of unpleasant sensory stimulation, but also associated with the salience network, were highly reactive but less modulated in female patients with MD. These results support and extent findings on altered processing of salience and of negative sensory stimuli even of a non-painful quality in female patients with MD.

**Electronic supplementary material:**

The online version of this article (10.1007/s00406-020-01123-0) contains supplementary material, which is available to authorized users.

## Introduction

Negative interpretation biases have been amply investigated for their suggested central role in depression [1]. Indeed, negative stimuli seem to be more salient for patients with major depression (MD) also on a neural level, as has been found in a number of neuroimaging studies [1]. Besides negative social and negative affective stimuli, unpleasant sensory stimulation has been investigated, mainly in the form of physical pain. Pain furthers avoiding damaging situations and maintaining homeostasis and can commonly be differentiated from other unpleasant sensory stimuli by its intensity. However, with regard to neural activation, painful stimuli show a high overlap with unpleasant sensory, or other negative salient stimuli [2, 3].

Recent studies focusing on the basic mechanisms of pain and unpleasant stimulation [4–11] indicate that patients with MD show decreased physical pain sensitivity as compared to healthy controls (HC) [5, 7, 12, 13] and increased pain and unpleasant stimulation thresholds were observed when pressure, thermal or electrical stimuli were applied to the skin [4, 14]. On the other hand, the perception of cold and warmth has been shown to be unaltered in patients with MD [15]. Further, inflammatory pathways and the hypothalamic–pituitary–adrenal (HPA) axis have been identified to play a role in the interaction between pain and depression [16, 17].

A vast array of neuroimaging studies in healthy subjects investigating the neural basis of physiological pain and unpleasant stimulus processing observed activation in the somatosensory cortex, the dorsolateral prefrontal cortex, the thalamus, the cingulate cortex and the bilateral insula under mildly painful stimulation, but also while being confronted with warm, but not painful stimuli [2]. However, only a part of this signature pattern could be associated specifically with the somatosensory component of the stimulation, including the primary and secondary somatosensory cortex and the posterior insula [2, 18, 19]. It has been suggested, that activation of posterior parietal and prefrontal cortices is associated with the cognitive processing of noxious information [18]. The other brain regions have been linked to higher-order modalities. Further, the anterior cingulate cortex (ACC) and anterior insula have been related to the processing of pain and unpleasantness [18, 20, 21], most likely coding the salience aspect of these stimuli [22].

The very few neuroimaging studies in MD investigating the underlying neural substrates of pain processing, as a type of unpleasant somatosensory stimulation, observed heterogeneous findings. An fMRI study investigating neural activations during the application of a mildly painful 45 °C heat stimulus in adult patients with MD revealed a relative hyperactivation of the prefrontal cortex during pain application, along with increased pain thresholds compared to HC [6]. Strigo et al. observed increased neural activation in the anterior insula, the right amygdala, and the dorsal anterior cingulate cortex only upon the expectation of painful heat stimuli as investigated in young adults with current depression by otherwise unaffected pain thresholds. This was interpreted as increased affective processing prior to the experience of the stimulus in MD [8, 9] and as a neural correlate of greater fear. In contrast, during the actual painful stimulation, MD patients showed relatively decreased neural activation in a network including the periaqueductal gray, rostral anterior cingulate and the prefrontal cortex [8].

Previous studies showed an altered neural response to negative, not necessarily painful stimuli within structures of the salience network in MD [1, 23]. Therefore, altered activation in the ACC and insula observed in depression might be related to differences in pain processing, but might also be due to different processing of the salience aspect of the stimulation. Moreover, some neuroimaging studies indicate fundamentally altered neural processing in MD within brain regions playing a crucial role in major depression, emotional salience and interoception, such as the anterior insula and the anterior cingulate cortex (ACC) that are also part of the pain processing network [24–28].

Based on these results regarding altered processing of painful but also salient stimuli in patients with MD, we investigated whether alterations within the network processing salient, unpleasant sensory stimuli would be observable even with non-painful sensory stimulation. We hypothesized that altered sensory stimulus processing would not only be evident at the high intensity of painful stimulation coming along with real or perceived tissue damage in depression but already at lower intensity unpleasant stimulation. Depression severity was assumed to correlate with alterations in brain activation. To elucidate these assumptions, we used parametric electric stimulation with increasing levels of unpleasantness during fMRI in patients with MD and HC.

## Materials and methods

### Subjects

54 young female subjects, aged 13–35 years (SD = 6), were included in this study. Due to technical problems causing incomplete acquisition of fMRI data in five cases, we had to exclude two subjects of the healthy controls (HC) and three subjects of the major depression (MD) group. Further, we had to exclude two subjects of the MD-group as they were not capable to differentiate stimulus intensities above chance level (accuracies of 25% and 5%). In total, 22 young adults with MD and 25 HC, 43 of them right-handed, were included in the final analyses. In detail, we investigated 18 subjects younger than 18 years of age, 9 of them diagnosed with MD. 9 subjects were between 18 and 21 years of age (1 diagnosed with MD) and 20 subjects were older than 21 years of age, 12 of them diagnosed with MD.The MD-group and HC were matched for age and education (see Table 1 and supplementary material section). Past and current psychiatric diagnoses were assessed using the Structured Clinical Interview for DSM-IV. Depression symptom severity was assessed by the Beck Depression Inventory (second edition, BDI-II [29];) in its German version [30]. HC had no current nor lifetime psychiatric diagnoses and served as control group. In the MD-group, besides dysthymia according DSM-IV in two subjects and a history of anorexia nervosa, currently recovered in one subject, no other current or lifetime psychiatric comorbidities were found.

**Table 1:** Summary of statistics of demographic, clinical and behavioral data in healthy controls (HC) and the major depression (MD) group

	HC		MD		*p* value	*T* value
	Mean	SD	Mean	SD		
Behavioral/Questionnaire data						
Age (years)	19.84	5.50	22.82	6.27	0.089	− 1.75
BDI	4.36	4.95	29.90	11.92	0.000	− 9.42
Electrical stimulation						
Stim. sensitivity: ratio: Stim. max./Stim. min. (Neuralect 2405 M)	4.43	2.74	4.34	2.21	0.929	− 0.45
Stim. sensitivity: ratio: Stim. max/Stim. min. (Neuralect 2406 M)	3.40	0.92	3.17	1.50	0.692	− 2.03
Stim. sensitivity: raw values: Stim. max./Stim. min. (Neuralect 2405 M)	13.17/4.40 *n* = 15	9.10/2.83	9.93/2.99 *n* = 12	6.36/1.41	0.305/0.127	5.43/8.57
Stim. sensitivity: raw values: Stim. max./Stim. min. (Neuralect 2406 M)	2.68/0.97 *n* = 10	2.78/0.93	2.23/0.96 *n* = 10	2.48/1.24	0.710/0.984	1.87/0.10
Stim. level identified (correct responses in %)	64	0.12	61	0.10	0.240	− 1.23

Statistical values for age, BDI and electrical stimulation are *t* values and stem from two-sided unpaired *t* tests

*Stim. max. *maximum of stimulus intensity level, *stim. min.* minimum of stimulation intensity level, *sd* standard deviation, *BDI* beck depression inventory

To account for gender differences and to reduce sample heterogeneity, only female participants were included. Patients were recruited from inpatient (*n* = 16) and outpatient units (*n* = 6) of the Department of Psychiatry and Psychotherapy and the Department of Child and Adolescent Psychiatry and Psychotherapy at Ulm University Hospital. All patients were in ongoing treatment. All gave written informed consent prior to the study, in the case of minors, written informed assent was given by participants as well as written informed consent by their caregivers. The study was approved by the local ethical committee of Ulm University and conducted in accordance with the Declaration of Helsinki. Participants with any severe medical disorder, epilepsy, substance use disorder and psychotic disorders were excluded from the study. To control for hormonal influences on neural processing, data were acquired within day 1 and day 10 after onset of menstruation (menstruation/follicular phase of menstural cycle) or after at least 14 days of continuous intake of oral contraception.

Smoking cigarettes regularly was reported by 3 subjects of the MD- and 4 subjects in the HC-group. In the HC-group, no data regarding smoking was available from 4 subjects. However, smoking was prohibited at least 2 h before fMRI-scanning. Antidepressant medication was not interrupted prior to scanning. 16 of the depressive patients took antidepressant medication which was held stable for at least 2 weeks before scanning (5 sertraline, 2 fluoxetine, 1 escitalopram, 1 citalopram, 1 mirtazapine, 2 venlafaxine, 1 escitalopram + bupropion, 2 sertraline + mirtazapine, 1 fluoxetine + mirtazapine). Any concomitant medication, i.e. topiramate, quetiapine and pregabalin, respectively in one subject each was discontinued for a wash-out phase of 3 days before fMRI scanning. None of the participants was on a regular medication with analgesics and no as needed medication was taken within 3 days before scanning.

Two-sided unpaired t-tests were computed to analyze psychometric scores.

### fMRI paradigm

Unpleasant physical sensations as a proxy for pain were induced via electric stimulation over the dorsum of the left hand as described in Adolph et al. [31]. The experimental unpleasant stimuli conformed to the guidelines for experimental pain (non-invasive, no tissue damage, avoiding movement, ethically acceptable, reproducible, physiologically relevant) as described previously [32]. Individual upper and lower boundaries of stimulus intensities were assessed prior to the functional scan. In a first step, the minimum stimulus intensity was assessed as the lowest level at which the subject could reliably perceive the unpleasant stimulus. One stimulus consisted of a train of four electrical square pulses with a duration of 1 ms each (100 Hz). Based on the minimum level (defined as level 1), the stimulation was increased stepwise to the individual maximum intensity that was perceived as unpleasant but not painful (defined as level 4). Intensity levels 2 and 3 were spaced equidistantly in-between levels 1 and 4. Subjects gave direct feedback and permission to increase stimulus intensity after each single step. All intensity levels were classified as unpleasant by the subjects. After the individual assessment, subjects were trained to correctly rate the stimulus levels 1–4 (1 = subjectively just detectable, 4 = subjectively clearly unpleasant, but well tolerable and not painful, 2 and 3 in between) with stimuli provided at random and were asked to rate stimulus intensities by pressing corresponding buttons on a four-button box during scanning. This procedure was repeated until subjects gave correct ratings for each stimulus. A total of 24 electrical stimuli (six per level) were administered during the scan, resulting in duration of about 10 min for the electrical stimulation task as described in Adolph et al. [31]. Participants were instructed to rate stimulus intensities during fMRI by pressing buttons on the same four-button box. Button presses and reaction times upon delivery of each electrical stimulus were recorded by a PC. Response times for button presses were not constrained. However, prior to each experimental session, subjects had been instructed to react as accurate and as fast as possible, balancing speed versus accuracy. A short signal tone of constant pitch (1000 Hz) and volume was delivered 1.5 s before electric stimulation to experimentally control for effects of expectation and attention. Trial order was pseudo-randomized in a way that one level of intensity appeared no more than twice in sequence. There were four parallel versions of the PC-controlled stimulation protocol with four different pseudo-randomized schemes of stimulus sequences. The average interstimulus interval across all versions was 24.3 s. Subjects were instructed to keep their eyes open during the entire session and to focus their view to the roof of the MR tunnel [31]. Due to technical conditions, we had to use different stimulus electrodes in adults (Neuralect 2405 M) and in minors (Neuralect 2406 M) that restricted the direct comparison of electrical stimulus intensity levels between groups (see supplementary material section for details on stimulus electrodes). Thus, ratios of maximum (level 4) and minimum stimulus intensities (level 1) were computed for each electrode to compare groups in this respect. Two-sided unpaired t-tests were computed to analyze the accuracy of stimulus ratings and intensity levels of electrical stimulation.

### Functional data acquisition

Functional imaging data were obtained using a 3 T MAGNETOM Allegra scanner (Siemens, Erlangen, Germany). High resolution anatomical T1-weighted images (1 × 1 × 1 mm^3^ voxels) were obtained [BW = 130 Hz/Pixel, repetition time (TR) = 2500 ms, inversion time (TI) = 1.1 s, echo time (TE) = 4.57 ms, flip angle = 12°] in each subject. For functional imaging, a T2*-sensitive gradient echo sequence was applied. 35 transversal slices were recorded at a TR of 2000 ms with an image size of 64 × 64 voxels. The field of view (FOV) was 230 mm, and slice thickness was 2.5 mm with an interslice gap of 0.5 mm. TE was 33 ms with a flip angle of 90°. Number of volumes during the unpleasant electrical stimulation task was 305. Before each scanning session, 6 images were acquired to allow for T1 saturation effects and discarded from further analysis.

### fMRI-data analysis

For image preprocessing we used Statistical Parametric Mapping (SPM12, Wellcome Department, London, UK) with a random effects model for group analyses. Data from each session were preprocessed including slice timing, realignment (translational: moving the image volume along an axis in space: *x*-, *y*-, and *z*-axes; rotation: turning the image around an axis in space: *x*–*y*, *x*–*z*, *y*–*z*; exclusion threshold: 1 mm/1°) and normalization into a standard template (Montreal Neurological Institute, MNI). Accordingly, coordinates of voxels are reported in MNI space. Smoothing was applied with an 8-mm FWHM isotropic Gaussian kernel. Intrinsic autocorrelations were accounted for by AR (1) and low frequency drifts were removed via high-pass filtering.

For individual first level analyses, a general linear model was used to estimate the height of neural activation associated with each of the four stimulus intensities. Onsets of individual trials for each of the four different intensity levels were modelled as stick functions and were convolved with the hemodynamic response function. Regressors representing the six motion parameters were additionally added to the design matrix and were integrated into the statistical analyses as were the onsets of the preceding warning tone and motor responses as regressors of no interest.

For second level group analyses, we computed a 2 × 4 ANOVA model with the factors ‘group’ (MD and HC) and ‘condition’ (four stimulus intensity levels). Increasingly unpleasant sensory stimulation was modeled using an equidistant, increasing contrast weight in each group: -3 -1 1 3. T-contrasts were then used to assess brain activation related to increasingly unpleasant stimulation separately in each group and over both groups and to compare effects of this stimulation between groups. Interaction contrasts (HC > MD, MD > HC) were masked by the t-contrast modeling increasing unpleasant stimulation levels over both groups, using the latter as an inclusive mask (*p* < 0.05 at voxel level). Analyses were conducted to test for differential effects (‘increasingly unpleasant stimulation’) between HC and the MD-group at a statistical threshold of *p* < 0.001 with a minimum cluster size of 80 voxels corresponding to a significance level of *p* < 0.05 corrected on the cluster level.

Further, to test on significant correlations between neural activations and depression scores but also age, we extracted parameter estimates from significant cluster activations. Hereby, the beta values for each level of unpleasant stimulation were extracted separately. Stimulation levels 1 to 4 were then weighted increasingly (-3 -1 1 3) and summed up. The weighted sum score was then used for the correlation. Correlations with BDI were calculated according to hypotheses. Correlations with age were calculated on an exploratory bases assuming that if brain activations indeed reflect effects of illness, these effects could be more marked with age in the MD group while no effects should be evident in controls. To control for multiple comparisons (parameter estimates from 3 brain regions), the nominal level of *p* < 0.05 was adjusted by mean false discovery rate computed according to Benjamini et al. [33] leading to a critical threshold of *p* < 0.0313.

## Results

### Demographic and behavioral data

HC and MD-group were roughly matched for age with a slightly but not significantly higher mean age in the MD-group. Both groups were matched for type of school attended currently or level of education completed. For further details on education please see the supplementary material section.

Regarding sensory stimulation, no group differences were found. Both groups identified the level of unpleasant stimulation intensity (1–4) in more than 60% of cases correctly with no differences regarding accuracy. None of the subjects confused levels 1 and 4. In only very rare cases levels 1 and 3 or levels 2 and 4 were mixed and the majority of mistakes involved mixing neighboring levels. Total scores and analyses of psychometric measurements are summarized in Table 1. For further details regarding absolute stimulus intensities of the four levels of both groups, see supplementary material section.

### fMRI data

**Fig. 1:**
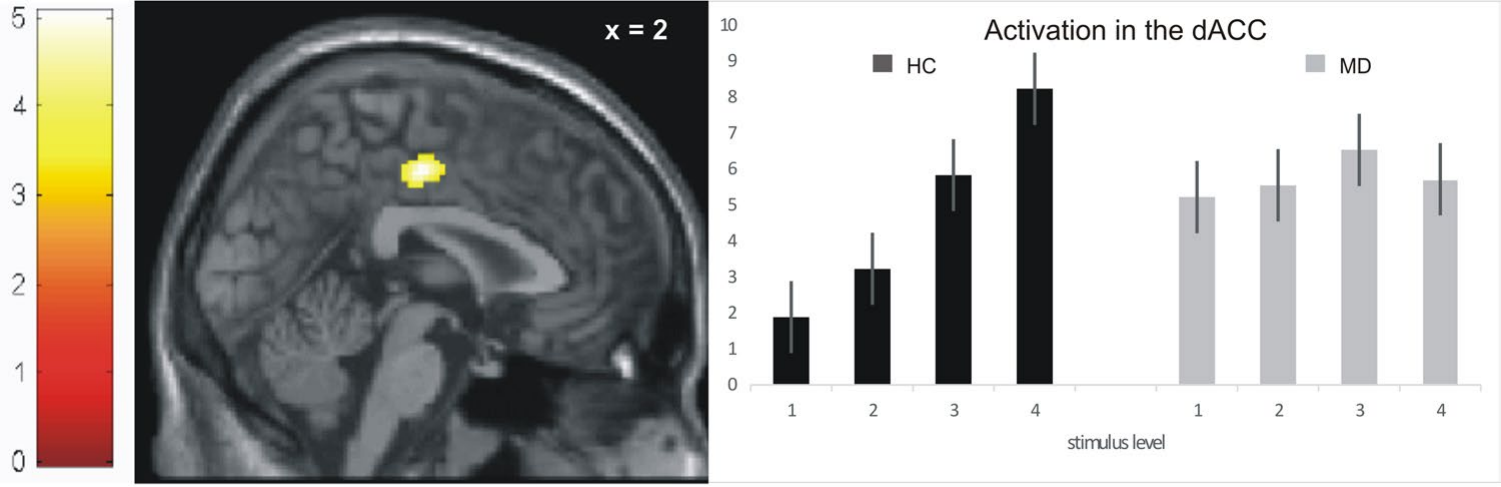
fMRI-results during electric stimulation with increasing levels of unpleasant sensory stimuli in major depression (MD) and healthy controls (HC). The brain slide depicts significant (*p* < 0.001, *k* > 80 Vx; *p* < 0.05 corrected on cluster level) results from between group analyses comparing differential (‘increasingly unpleasant stimulation’ condition with 4 levels of increasingly unpleasant electric stimulation) neural activations between HC > MD during fMRI. Bar charts show fMRI parameter estimates extracted from peak voxel activation within the dACC (dorsal anterior cingulate cortex) with standard error of the mean

The t-contrast modelling increasingly unpleasant stimulation over both groups confirmed the expected network of brain regions including the dorsal anterior cingulate cortex (dACC)/ supplementary motor cortex (SMA) (peak voxel *x*/*y*/*z* = 2/−16/46, *Z* = 6.41), the right primary somatosensory cortex (S1) (peak voxel *x/y/z* = 40/−24/54, *Z* = 6.20), the right and left posterior insula (pI) (peak voxel *x*/*y*/*z* = 48/−22/18, *Z* = 4.98; peak voxel *x*/*y*/*z* = −48/−30/20, *Z* = 4.51), the amygdala (peak voxel *x*/*y*/*z* = −36/2/−14, *Z* = 4.10) and cerebellum (peak voxel *x*/*y*/*z*  = 24/−40/-−28, *Z* = 5.00; peak voxel *x*/*y*/*z* = −24/−40/−28, *Z* = 5.00) at *p* < 0.05 corrected at the cluster level. Analyses of interaction effects to investigate group differences regarding the ‘increasingly unpleasant stimulation’ condition revealed significant effects in the dACC, S1 and posterior insula (F-contrast). A t-contrast confirmed significantly enhanced neural activation within the S1, pI and the dACC/ SMA in HC as compared to MD (see Table 2 and Fig. 1). According to Wager et al. [2], these regions can be taken as part of the common network with a reactivity to physically painful/unpleasant stimuli as compared to non-painful sensory stimulation. Within these brain regions, HC showed the expected parametric increase of neural activation with increasing stimulus intensity in all of the regions. In the MD-group, the fMRI signal differentiated to a significantly smaller extent between stimulus intensities as compared to HC (see Fig. 1) were evident. None of the brain regions showed decreased activation in HC compared to MD for this contrast. Figure S1 (see supplementary material) shows effects of increasing unpleasant stimulation separately for each group and for group comparisons over the whole brain.

**Table 2:** Significant (*p* < 0.001, *k* > 80 Vx; *p* < 0.05 corrected on cluster level) results from between group analyses comparing differential neural activations for the 'increasingly unpleasant stimulation' contrast between HC = healthy controls and MD = major depression group upon electrical stimulation during fMRI

BA	Anatomic label	HC > MD						
		Side	Cluster size	MNI				
		*L/R*	NV	*x*	*y*	*z*	*Z*	*t*
24	Dorsal anterior cingulate cortex (dACC)/supplementary motor area (SMA)	R	383	4	− 16	46	4.93	5.17
3	Primary somatosensory cortex (S1)	R	219	40	− 24	54	4.89	5.13
	Posterior insula (pI)	R	90	40	− 16	− 2	3.90	4.02

*BA* Brodman area, *L *left, *R *right, *NV *number of voxels, *MNI *Montreal Neurological Institute (*x*-, *y*-, *z*-coordinates are provided in mm), *Z* *Z* value, *t* *t* value

To control for sample inhomogenities regarding age, medication and handedness, we reanalyzed a highly homogenous subsample of our data including only right handed, medicated patients aged 18 or older with no comorbidity additional to major depression (*n* = 12) and compared them with the group of right-handed healthy controls aged 18 or older (*n* = 15). Using the same 2 × 4 ANOVA model with the factors ‘group’ (MD and HC) and ‘condition’ (four stimulus intensity levels), t-contrasts modelling interaction effects revealed significantly enhanced neural activation in HC as compared to MD within the S1, pI and the dACC/SMA (for details see supplementary table). No significant effects were found for MD > HC. Thus, the analysis in the more homogenous subsample confirmed findings in the larger but more heterogeneous whole sample supporting the validity of our results.

### Correlation analyses

**Fig. 2:**
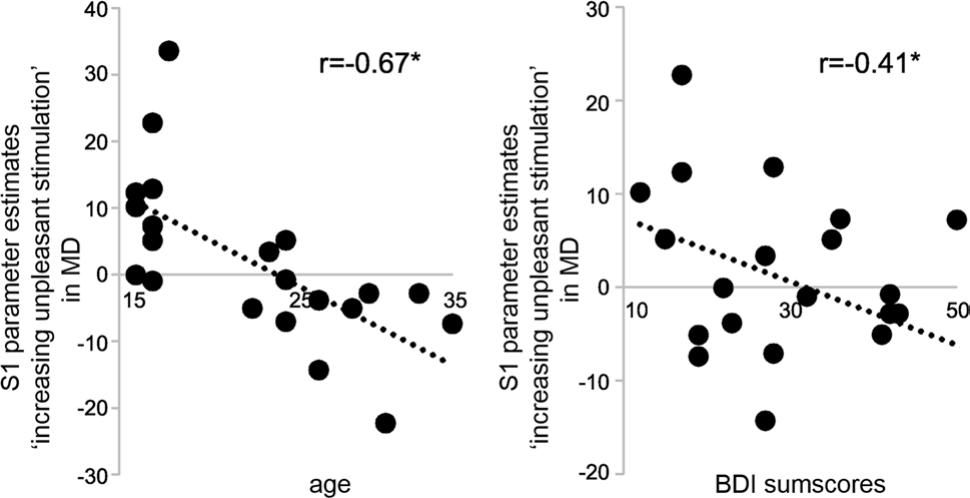
Significant correlations between fMRI effects related to increasingly unpleasant electric stimulation, age and individual BDI-sumscores. The scatter plots depict the significant (significance was defined as p < 0.0313 after correction according to Benjamini et al. [33]) correlation between mean individual parameter estimates of modelled fMRI effects related to increasingly unpleasant sensory stimulation within the primary somatosensory cortex (S1) cluster and individual BDI-sumscores (left panel) as well as age (right panel) in patients diagnosed with major depression (MD). The dotted line represents the trendline

To test on correlations between neural reactivity to unpleasant/painful stimulation (contrast: ‘increasingly unpleasant stimulation’) with depression scores according to our hypothesis, we extracted parameter estimates of the three significant cluster activations revealed by between group analyses. Regarding the S1, differential neural activation (‘increasingly unpleasant stimulation’) was significantly and negatively correlated with BDI-sumscores (*r* = −0.41; *p* = 0.029, corrected for multiple comparisons) in MD, indicating that those MD patients with lower S1 activation had a higher depressive symptom severity (Fig. 2). Further, significant (corrected for multiple comparisons) negative correlations with age were observed for neural activations within the S1 (*r* = −0.67, *p* = 0.000), dACC (*r* = −0.51, *p* = 0.015) and pI (*r* = −0.44, *p* = 0.020) in MD (Fig. 2), indicating that older MD patients differentiated less between stimulus intensities. In the control group, no significant correlations were found for BDI nor age (S1-BDI (*r* = 0.15, *p* = 0.238), S1-age (*r* = 0.03, *p* = 0.443), dACC-BDI (*r* = 0.02, *p* = 0.462), dACC-age (*r* = −0.08, *p* = 0.351), pI-BDI (*r* = −0.03, *p* = 0.443) and pI-age (*r* = −0.18, *p* = 0.194)) and the parameters BDI and age themselves were not significantly correlated in neither group (MD: *r* = 0.29, *p* = 0.095; controls: *r* = 0.19, *p* = 0.181).

## Discussion

In the current study, females with MD showed a flattened modulation in response to increasing unpleasant electric stimulus intensity in the dorsal anterior cingulate cortex (dACC), the primary somatosensory cortex (S1), and the posterior insula (pI). The findings suggest altered processing of unpleasant sensory stimuli in networks related to somatosensory and affective processing of pain and saliency. These results can extend the—so far very limited—knowledge on neural processing of negative salient sensory stimuli in MD and may suggest that this altered processing is not restricted to high intensity, painful sensory stimulation.

The network including dACC, S1 and pI where we found relatively decreased activation in female MD compared to HC has been previously related to as the experience of unpleasant and painful stimuli [2]. These areas showed an increasing activation related to increasing stimulus intensity irrespective of group. However, females with MD showed a still detectable but significantly flattened modulation in those areas. This effect correlated with increasing depressive symptom severity, particularly in the S1 and also with age in all three brain regions. While the correlation with depressive symptoms seems to more clearly point towards a relationship of clinical symptomatology and neural processing of acute pain, the correlational findings with age have various possible explanations. Although age and current depression severity were not directly correlated, effects of illness may have been more marked in young adults than in adolescents. Although this was not assessed systematically, older subjects in our sample had longer histories of illness and also a longer history of treatments than younger ones. Furthermore, medication was more common in older than in younger subjects and more common in the more severely ill ones. Therefore, an effect of medication cannot be ultimately ruled out. Studies in healthy subjects point towards a decreased neural reactivity of related brain areas after intake of antidepressants [34, 35]. Our findings of decreased activation of these areas are in line with the findings of Rodriguez et al. [10], also investigated predominantly medicated patients using repetitive heat stimuli. These findings are in contrast to a study by Baer and colleagues [6], who found similarly modulated activation in the ACC and the insula in participants with MD as compared to HC during parametrically increasing stimuli intensities with heat pain in non-medicated patients. This could be another hint for a potential role of the medication. On the other hand, another study by Baer and colleagues [5] did not find specific effects of SSRI treatment on the neural processing of unpleasant sensory stimulation in patients with MD. Still, results of this study should be interpreted with having this possible limitation in mind.

The S1 and the pI have been related to the somatosensory processing of physical pain [2]. Decreased modulation in those areas in patients with MD might point towards a dysfunctional processing of unpleasant haptic sensations, i.e. all intensities were of similar, and maybe even increased salience. This notion is supported by our observation that neural reactivity of these regions was rather increased for the low unpleasant sensory stimuli in MD. This finding could be related to clinical reports of patients with MD often reporting multiple pain complaints [36] and negative stimuli being more salient [2]. As a possible mechanism, all negative somatosensory stimuli (despite their objective intensity) might be processed with the same, relatively increased, activation. In addition, the finding is in line with the interpretation of Rodriguez et al. [10] who suggested that aberrant activation of somatosensory cortices in MD upon acute pain application could relate to the inability of depressed patients to identify the turning point between warm sensation and heat pain in their study.

Interestingly, in a study comparing the processing of unpleasant electric stimulation in participants with previous non-suicidal self-injury (NSSI) to HC, activation of the pI was similar in both groups, while participants in the NSSI group showed a flattened response in the anterior insula, which is known to play a role in affective pain processing [3]. Similarly, in our study, participants with MD showed a flattened modulation in the dACC, a region known to be involved in the affective processing of pain [21]. It is important to note that participants with MD did not show a decreased activation in the dACC per se, but that activation did not differentiate across increasing stimuli, showing rather high activation even during very low stimulus intensities. In another study, similarly unmodulated activation in areas involved in the affective processing of pain (i.e. the rostral ACC) was found in patients with MD during heat stimulation [8]. This is in line with findings by Strigo and colleagues [37], who found that young depressed adults rated non-painful heat stimuli as unpleasant significantly earlier than HC, calling this effect ‘emotional allodynia’. Emotional allodynia can be understood as a negative affective response to per se not painful stimuli. High negative affective response, in line with high dACC activation during minor electric stimuli, might be related to more frequent multiple pain complaints in MD [36]. As the ACC and insula are part of the saliency network, results of our study might also point towards an overall increased processing of negative salience in patients with MD, as noted in previous studies [1].

Besides potential influences of medication as discussed above, as a second limitation, we had to change the stimulation electrode during the course of the experiment due to malfunctioning. For both electrodes (Neuralect 2406 M and 2405 M), the same parameters of stimuli were used and differences in stimulus intensities were identified with equal accuracy with both devices. However, measures of absolute sensory stimulation thresholds differed between electrodes which prevented us from using electrical stimulation thresholds for further analyses. Heterogeneity regarding brain maturation over our sample of postpubertal adolescents and young adults is a potential source of bias and increases variance of the sample. Accordingly, results in the more homogeneous subsample of participant aged 18 or older showed rather higher significance levels. Recruitment of only females restricts the interpretability of our results. Measurement in both participants with and without taking a birth control pill could further have biased our findings. However, a meta-analytic review of pain perception across the menstrual cycle suggested influences of sex hormones when applying electric stimulation, but effect sizes were small to moderate [38]. Inclusion of 4 left-handed subjects, 2 subjects with a co-diagnosis of dysthymia and one subject with a history of remitted anorexia are further potential limitations of our study. Although the stimuli applied were suited to activate the brains pain processing network, the use of unpleasant but not painful sensory stimuli restricts the interpretability of the results.

In the current study, female patients with MD showed a flattened modulation in the processing of increasingly unpleasant electric stimuli. This was evident for brain areas involved in the somatosensory, as well as in the affective processing of physical pain, unpleasant sensory stimulation and saliency. While the identification of different levels of stimulation intensity remained intact, neural processing did not differentiate between stimuli. Moreover, low-level non-painful stimuli elicited relatively high activations, similar to indeed unpleasant electric shocks. Results of this study suggest a clearly aberrant neural processing of unpleasant sensations in female MD, possibly through increased saliency of negative stimuli or through actual changes in the processing of sensory stimulation even of a non-painful quality. The results may for example help to elaborate new hypotheses for example prima-vista contradictory findings of hyperalgesia and elevated pain thresholds in MD.

## Electronic supplementary material

Below is the link to the electronic supplementary material.

Supplementary file1 (DOCX 136 kb)
