# Supplementary material for: Differential neural processing of unpleasant sensory stimulation in patients with major depression
Source: Eur Arch Psychiatry Clin Neurosci. 2020 Apr 11;271(3):557–65. doi: 10.1007/s00406-020-01123-0 (PMC7981307; doi:10.1007/s00406-020-01123-0)
Supplement: Supplementary file 1 — Supplementary file1 (DOCX 136 kb) [file 406_2020_1123_MOESM1_ESM.docx]

**Supplements**

**Differential neural processing of unpleasant sensory stimulation in patients with major depression**

Kathrin Malejko^1*^, Rebecca C. Brown^2*^, Paul L. Plener^2,3^, Martina Bonenberger^2^, Heiko Graf^1^, Birgit Abler^1^

^1^ Ulm University, Department of Psychiatry and Psychotherapy III, Ulm, Germany

^2^ Ulm University, Department of Child and Adolescent Psychiatry and Psychotherapy, Ulm, Germany

³ Department for Child and Adolescent Psychiatry, Medical University of Vienna, Vienna, Austria

Methods

2.2. fMRI paradigm - electrical stimulation

We used an universal size standard grounding pad (Ambu® Australia Pty Limited; Neutralect 2405M) for adult MD- and HC patients. For the minors, we used an universal size split grounding pad (Ambu® Australia Pty Limited; Neutralect 2406M). Although the conductive areas between these grounding pads differed only slightly (2405M: 10200 mm2; 2406M: 10338 mm2), we had to apply higher amplitudes with the new pads to overcome perception thresholds, presumably due to the split (within one grounding pad) and an increase in electrical resistance. This restricted a direct comparison between electric currents or amplitudes. However, both electrodes equally and reliably elicited unpleasant sensations in the range from subjectively just detectable to subjectively clearly unpleasant, but not painful and thus for focusing on the intended sensory-discriminative feature of a parametrically increase in stimulus intensities that were individually adjusted.

Results

3.1. Demographic and behavioral data

School types: Hauptschule: attends/attended school that does not terminate with a secondary school level-I certificate (HC: n=4, MD: n=4); Realschule: attends/attended school that terminates with a secondary school level-I certificate (HC: n=8, MD: n=7); Gymnasium: attends/attended school that terminates with the general qualification for university entrance (HC: n=13, MD: n=11).

The mean stimulus intensity in the MD group of level 1 was 2.07 mA (standard deviation (sd)=1.66), for level 2 the mean stimulus intensity was 3.49 mA (sd=3.01), for level 3 4.92 mA (sd=4.54) and for level 4 6.43 mA (sd=6.26). For HC, the mean stimulus intensity of level 1 was 3.03 mA (sd=2.82), for level 2 4.96 mA (sd=4.72), for level 3 6.90 mA (sd=6.72) and for level 4 8.84 mA (sd=8.76).

**Figure S1**

Results of t-contrasts conducted to test for differential parametric effects (increasing unpleasant stimulus intensity levels) in patients with depression (MD) and healthy controls (HC) separately and comparing the two groups (interaction effects).

**
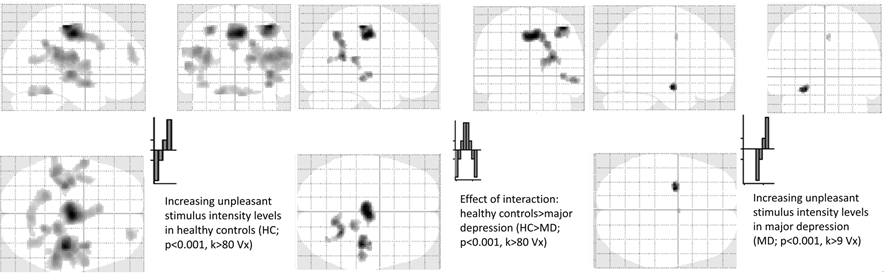
**

**Supplementary Table**

|  |  | *HC>MD* | | | | | |  |
| --- | --- | --- | --- | --- | --- | --- | --- | --- |
| *BA* | *Anatomic label* | *Side* | *cluster size* | *MNI* | | |  |  |
|  |  | *L/R* | *NV* | *x* | *y* | *z* | *Z* | *t* |
| 24 | dorsal anterior cingulate cortex (dACC)/supplementary motor area (SMA) | R | 574 | 4 | -12 | 46 | 5.74 | 6.45 |
| 3 | somatosensory cortex (S1) | R | 382 | 42 | -24 | 54 | 6.93 | 8.24 |
|  | posterior insula (pI) | R | 368 | 46 | -28 | 18 | 5.06 | 5.55 |

Significant (p<0.001, k>200Vx; p<0.05 FWE corrected on cluster level) results from between group analyses comparing differential (‘increasingly unpleasant stimulation’) neural activations between HC=healthy controls>MD=major depression upon electrical stimulation during fMRI for the subgroup (HC: n=15, MD: n=12) of righthanded subjects aged 18 or older, all medicated with antidepressants.

BA=Brodman area; L=left; R=right; NV=number of voxels; MNI=Montreal Neurological Insitute (x-, y-, z-coordinates are provided in mm), Z=Z-value, t=t-value
